# Elevated diastolic blood pressure until mid-gestation is associated with preeclampsia and small-for-gestational-age birth: a population-based register study

**DOI:** 10.1186/s12884-019-2319-2

**Published:** 2019-05-28

**Authors:** J. Gunnarsdottir, T. Akhter, U. Högberg, S. Cnattingius, A. K. Wikström

**Affiliations:** 1 Department of Women’s and Children’s Health, Uppsala University, SE-75185 Uppsala, Sweden; 2 Clinical Epidemiology Unit, Department of Medicine Solna, Karolinska Institutet, Stockholm, Sweden

**Keywords:** Blood pressure, Preeclampsia, Foetal growth restriction, Small-for-gestational-age

## Abstract

**Background:**

Gestational hemodynamic adaptations, including lowered blood pressure (BP) until mid-gestation, might benefit placental function. We hypothesized that elevated BP from early to mid-gestation increases risks of preeclampsia and small-for-gestational-age birth (SGA), especially in women who also deliver preterm (< 37 weeks).

**Methods:**

In 64,490 healthy primiparous women, the change in systolic and diastolic BP from early to mid-gestation was categorized into lowered (≥ 0 mmHg decreased), and elevated (≥ 1 mmHg increase). Women with chronic hypertension, chronic renal disease, pre-gestational diabetes and systemic lupus erythematosus were excluded. Risks of preeclampsia and SGA birth were estimated by logistic regression, presented with adjusted odds ratio (aOR) and 95% confidence intervals (CI). Further, the effect of BP change in combination with stage 1 hypertension (systolic BP 130–139 mmHg or diastolic BP 80–89 mmHg) in early gestation was estimated.

**Results:**

Compared to women with lowered diastolic BP from early to mid-gestation, those with elevated diastolic BP had increased risks of preeclampsia (aOR: 1.8 [1.6–2.0]) and SGA birth (aOR: 1.3 [1.2–1.5]). The risk estimates were higher for preeclampsia and SGA when combined with preterm birth (aORs: 2.2 [1.8–2.8] and 2.3 [1.8–3.0], respectively). The highest rate of preeclampsia (9.9%) was seen in women with stage 1 hypertension in early gestation and a diastolic BP that was elevated until mid-gestation. This was three times the risk, compared to women with normal BP in early gestation and a diastolic BP that was decreased until mid-gestation. The association between elevated systolic BP from early to mid-gestation and preeclampsia was weak, and no significant association was found between changes in systolic BP and SGA births.

**Conclusion:**

Elevated diastolic BP from early to mid-gestation was associated with increased risks of preeclampsia and SGA, especially for women also delivering preterm. The results may imply that the diastolic BP starts to increase around mid-gestation in women later developing placental dysfunction disorders.

## Background

In pregnancies complicated with placental dysfunction disorders (i.e., preeclampsia and birth of small-for-gestational-age (SGA) infants), the blood pressure (BP) is generally higher in early gestation than in uncomplicated pregnancies [1–3]. Normal pregnancy is associated with gestational hemodynamic adaptations that include BP changes [4–7]. BP usually decreases from early to mid-gestation (“mid-gestational BP drop”), and this adaptation might benefit placental perfusion [8]. An absence of mid-gestational BP drop is associated with preeclampsia, but it is less clear whether this absence may also be relevant to SGA births [4, 9–11]. In normal pregnancy, BP usually increases from mid-gestation and onwards to reach pre-pregnancy levels in late gestation [4–6]. In women developing placental dysfunction disorders, systolic and diastolic BP increases faster than normal after mid-gestation [9–11].

We hypothesized that an increase in BP before mid-gestation may also be associated with higher risks for preeclampsia and SGA births, independently of the early-gestation BP. We further hypothesized that the association may be especially strong in women with preeclampsia and SGA who also deliver preterm (< 37 weeks).

In this population-based cohort study of 64,490 healthy primiparous women, we estimated the association between the change in systolic and diastolic BP from early to mid-gestation and the risks of preeclampsia and birth of SGA infants.

## Methods

### Data sources

Data were obtained from the Swedish population-based Stockholm-Gotland Obstetric Database, [12], which is based on the medical record system used in the region for all antenatal, delivery and postnatal care units. Data from the medical record system are forwarded daily to the database, which contains information from 2008 and onwards. In Sweden, antenatal and delivery care is standardized and free of charge. Home deliveries are rare. During the first antenatal visit, standardized to take place around gestational week 10, the mother is interviewed about her medical and reproductive history and smoking habits. Information on the number of prior misscarriages, pregnancies by assisted reproductive techniques in the index pregnancy and the presence of pre-gestational hypertension, diabetes and systemic lupus erythematosus (SLE) is registered in check boxes. The mother’s height is generally self-reported, while weight and BP are measured by the midwives and recorded in the database. A urine test is acquired for a dipstick test of proteinuria. In cases of a positive dipstick, another dipstick test is taken on a separate occasion within a short time. The BP is re-measured at each antenatal visit. The second visit is standardized to occur at 20–25 gestational weeks but occurs earlier if considered necessary. BP is measured in a sitting position, using manual BP equipment with a cuff size appropriate for arm circumference. Korotkoff V is used to measure the diastolic BP. At discharge from the hospital after delivery, the responsible doctor records complications during pregnancy and delivery, according to the International Classification of Diseases, tenth revision [ICD-10].

### Study population

**Fig. 1:**
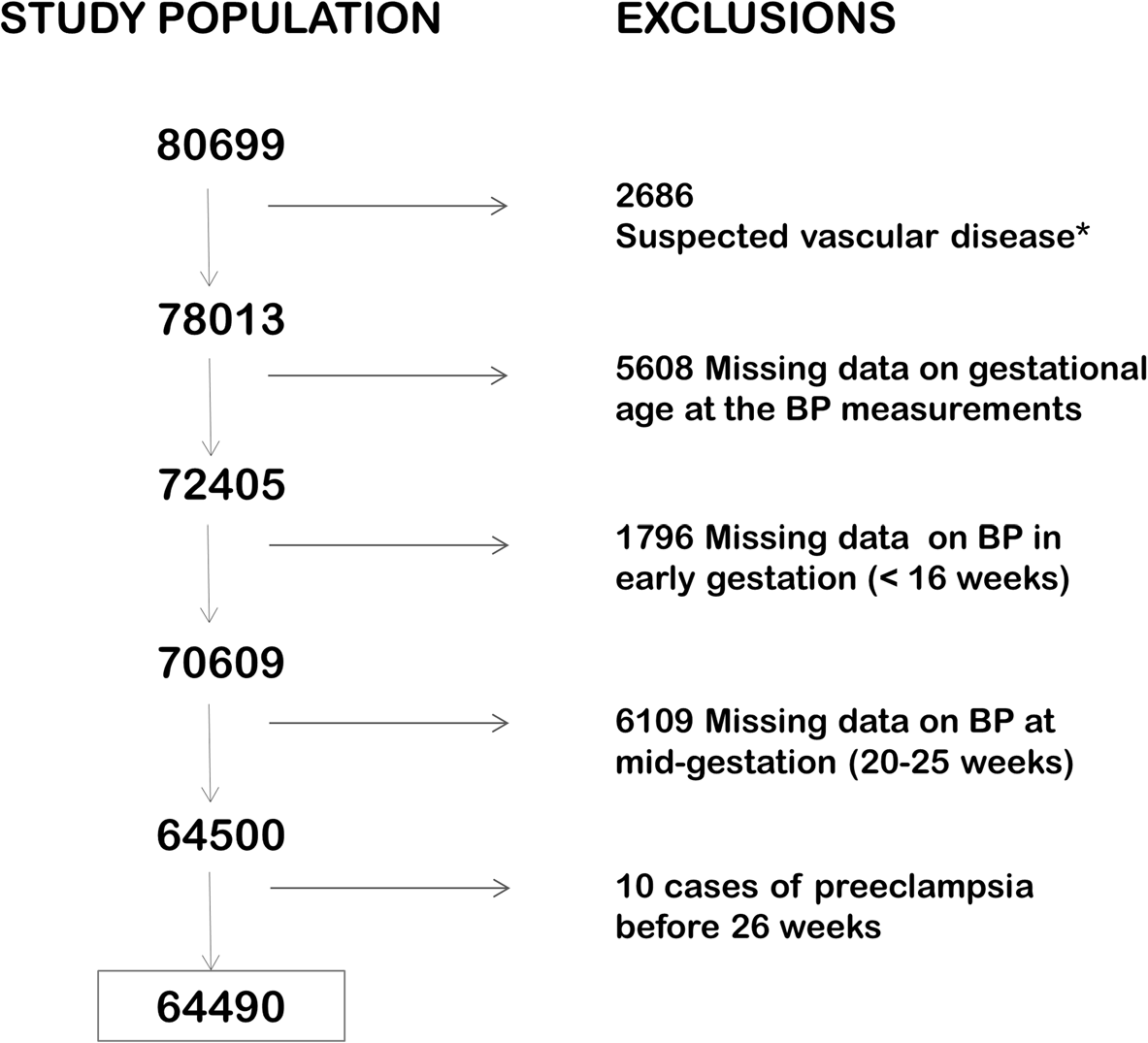
Flow chart of the study population that includes healthy primiparous women with singleton pregnancies. We excluded women with suspected vascular disease in early gestation,defined as one of the following conditions: *chronic hypertension*, identified as systolic blood pressure (BP) ≥140 or diastolic BP≥90 or BP medication in early gestation, registration of chronic hypertension by checkbox at the first antenatal visit or a corresponding diagnostic code after delivery; or *chronic renal disease*, identified by proteinuria before 20 gestational weeks (2+ on dipstick or 1+ on two consecutive occasions) or a diagnostic code for chronic renal insufficiency after delivery; or *pre-gestational diabetes*, identified by registration in a checkbox at first antenatal visit or a corresponding diagnostic code after delivery; or *systemic lupus erythematosus* as registered in a check box or a corresponding diagnostic code after delivery

The population was defined as healthy primiparous women who gave birth to a singleton from 2008 to 2014 in Stockholm or Gotland counties (Fig. 1). The risk of recurrence of placental dysfunction disorders is substantial [13–15]. Information on the outcome of previous pregnancies was lacking in this data, and therefore only primiparous women were included. Of 80,699 primiparous women, we excluded 2686 women with suspected vascular disease in early gestation, defined as one of the following conditions: chronic hypertension, chronic renal disease, pre-gestational diabetes or SLE. Chronic hypertension was defined by systolic BP ≥ 140 mmHg or diastolic BP ≥ 90 mmHg or blood pressure medication at the first antenatal visit, registration in a check box by the midwife at first antenatal visit, or by ICD-10 codes O10 and O11 at discharge from the hospital after delivery. Chronic renal disease was identified by ICD-10 code N18 (chronic renal insufficiency) or proteinuria within the first 20 weeks of gestation, defined as 2+ on a dipstick or 1+ on two separate occasions (more than 4 h apart). Women with pre-gestational diabetes were identified by a check box, as well by ICD-10 codes O24.0 and O24.3. Women with SLE were identified by a check box and by ICD-10 codes M32.1, M32.8 and M32.9. The study population was further confined to 64,500 women with a first registered BP measurement before gestational week 16 (“early gestation”) and a BP measurement between gestational weeks 20 and 25 (“mid-gestation”). The mean gestational age at the BP measurements was 10 weeks in early gestation and 22 weeks at mid-gestation. Finally, we excluded 10 women who developed preeclampsia before 26 weeks of gestation because this was within the period of defined exposure variable (see below). The final study population included 64,490 women (Fig. 1).

### Exposure

The main exposure was the change in the systolic and diastolic BP from early to mid-gestation. We calculated this by subtracting the mid-gestation measurements from the early-gestation measurements of systolic and diastolic BP separately. The change in BP was categorized as follows: 1) Lowered BP was defined as no change or a decrease by 1 mmHg or more, and 2) elevated BP was defined as an increase by 1 mmHg or more in BP between early to mid-gestation.

### Outcomes

Outcomes were preeclampsia and SGA birth, where the latter was used as a proxy for foetal growth restriction. Preeclampsia was defined through the ICD-10 codes O14-O15. According to Swedish national guidelines during the study period, the clinical definition of preeclampsia was a BP ≥ 140 systolic or ≥ 90 mmHg diastolic, measured on two separate occasions, combined with proteinuria (≥ 0.3 g/24 h or + 1 or more on dipstick and on at least one subsequent occasion). Preeclampsia diagnosis registered in the Nordic countries was previously retrospectively validated with the above definition as the gold standard, suggesting a positive predictive value of around 80% [16, 17]. An SGA infant was defined as a liveborn infant with a birth weight of more than two standard deviations below the mean weight for gestational age, according to the Swedish sex-specific foetal growth curve [18]. If preeclampsia or SGA was present in combination with preterm birth (< 37 weeks of gestation), the outcomes were defined as preterm. Gestational age was determined using the following hierarchy: a) date of embryo transfer (3.0%), b) early second trimester ultrasound (95.2%), c) date of last menstrual period (1.8%), and d) a postnatal assessment (< 1%). In the final cohort 2241 (3.5%) women developed preeclampsia, and 1936 (3.0%) women gave birth to an SGA infant.

### Covariates

The following covariates were considered as possible confounders: maternal early-gestation BP, body mass index (BMI) in early pregnancy, age at delivery, height, in vitro fertilization (IVF), prior recurrent misscarriages (three or more self-reported miscarriages), cohabitation status with partner, smoking in early pregnancy, and country of birth. The choice of covariates was based on previous reports of associations [19–23]. The covariates were categorized as shown in Table 1.

**Table 1:** The rate of preeclampsia and giving birth to a small-for-gestational-age (SGA) infant by maternal characteristics

Maternal characteristics	Numbers	Preeclampsia		SGA births	
		%	*p*-value	%	*p*-value
BP^a^ early gestation			< 0.001		0.049
Normal	56,491	3.0		3.0	
Stage 1 hypertension^b^	7996	7.0		3.4	
BMI^c^ early gestation (kg/m^2^)			< 0.001		< 0.001
< 18.5	1994	2.7		4.8	
18.5–24.9	44,024	2.9		3.0	
25–29.9	11,473	4.5		2.7	
≥ 30	4025	7.1		3.3	
Missing	2974	3.3		3.2	
Age (years)			< 0.001		< 0.001
< 25	9144	3.3		3.0	
25–29	20,368	3.4		2.7	
30–34	23,763	3.2		2.9	
≥ 35	11,199	4.4		3.8	
Missing	16	6.3		6.3	
Height (cm)			< 0.001		< 0.001
< 164	20,609	3.8		4.5	
164–171	28,915	3.5		2.6	
≥ 172	14,477	2.9		1.7	
Missing	489	2.9		3.5	
Cohabitation status			0.975		0.105
Living with partner	58,990	3.5		3.0	
Not living with partner	4815	3.5		3.6	
Missing	685	3.5		3.4	
In vitro fertilization			0.003		0.320
Yes	4437	4.3		3.2	
No	60,053	3.4		3.0	
Prior miscarriages (number)			0.022		0.022
≤ 2	63,822	3.5		3.0	
≥ 3 (recurrent)	668	5.1		4.0	
Smoking^d^			0.088		< 0.001
Yes	2626	2.7		5.2	
No	61,330	3.5		2.9	
Missing	534	3.6		2.6	
Country of birth			< 0.001		< 0.001
Sweden	41,392	3.7		2.6	
Other Nordic countries	875	3.9		3.0	
Outside Nordic region	12,551	2.8		4.3	
Missing	9672	3.4		3.3	

^a^BP: Blood pressure. ^b^Stage 1 hypertension, in early gestation: Systolic BP 130–139 mmHg or diastolic BP 80–89 mmHg before 16 weeks, ^c^*BMI* Body mass index, ^d^Daily smoking in early gestation. *P*-values were calculated with chi-square test

### Statistical analysis

The rate of preeclampsia and SGA was compared through different categories of the covariates in Table 1, and a chi-square test provided *p*-values. The risks of preeclampsia and SGA were calculated for women with elevated BP from early to mid-gestation, using women with lowered BP as the reference category. Analyses of the changes in systolic and diastolic BP were done separately. Odds ratios (ORs) with 95% confidence intervals (CI) were calculated, using unconditional logistic regression analysis. Adjustments were made for early gestation mean arterial blood pressure (MAP), maternal BMI, age, recurrent miscarriages, smoking, and country of birth. Early gestation MAP was used as a continuous variable, but the other variables were used in categories according to Table 1. Observations with missing values were excluded from the adjusted analysis. The covariates IVF and cohabitation status were omitted from the multiple regression models because these covariates were not independently associated with the outcomes (*p*-values > 0.05). Furthermore, maternal height was not included as this covariate is strongly correlated with BMI. The analyses of the risks of preeclampsia and SGA included women with both outcomes combined. To further investigate independent associations, we repeated the analysis of SGA in women who did not develop preeclampsia.

Further, the effect of the change in BP from early to mid-gestation was investigated in relation to the level of early-gestation BP that was dichotomized into normal BP (systolic BP <  130 mmHg and diastolic BP < 80 mmHg) and stage 1 hypertension (systolic BP 130–139 mmHg or diastolic BP 80–89 mmHg) [23]. These binary covariates were combined to create four groups: 1) Women with normal early-gestation BP and lowered BP towards mid-gestation, 2) Normal early-gestation BP and elevated BP towards mid-gestation, 3) Stage 1 hypertension in early gestation and lowered BP towards mid-gestation and 4) Stage 1 hypertension in early gestation and further elevated BP towards mid-gestation. The risk of preeclampsia was estimated using group 1 as the reference.

Lastly, the analysis was repeated for preterm preeclampsia and SGA, defined as preeclampsia or SGA birth in combination with preterm birth (< 37 weeks). The risks of preeclampsia and SGA were calculated for women with elevated diastolic BP from early to mid-gestation, using women with lowered diastolic BP as the reference category, and the same adjustments as above. All analysis was performed using Statistical Analysis Software version 9.3 (SAS institute, Inc., Cary, NC).

## Results

In the final cohort, 3.5% were diagnosed with preeclampsia. In women with stage 1 hypertension in early gestation and in obese women (BMI ≥ 30), the rate of preeclampsia was 7% (Table 1). Women aged 35 years or older were more likely to be diagnosed with preeclampsia than younger women, and preeclampsia occurred more often in short women (< 164 cm) than in tall women (≥ 172 cm). Preeclampsia occurred more often in women that conceived by in vitro fertilization than in spontaneously pregnant women, and the rate was also higher in women with a history of recurrent miscarriages (three or more) than in women without recurrent miscarriages.

Three percent of the women gave birth to SGA infants. Preeclampsia coincided with SGA in 290 of 1936 women who gave birth to SGA infants (data not shown in table). There was only a marginal difference in SGA rates between women with normal BP and stage 1 hypertension in early gestation (Table 1). Birth of an SGA infant was more likely in underweight women (BMI < 18.5) than in women of normal weight. Women aged 35 or older were more likely to give birth to SGA infants than younger women. The SGA rate was 4.6% in short women and 1.7% in tall women. Women with a history of recurrent miscarriages had higher rates of SGA than women without recurrent miscarriages.

In the excluded group of women with suspected vascular disease, 12.3% developed preeclampsia, and 4.6% gave birth to SGA infants (data not shown in table).

The association between changes in systolic BP from early to mid-gestation and preeclampsia seemed weak (Table 2), and there was no association between changes in systolic BP and SGA births (Table 3).

**Table 2:** Risk of preeclampsia by changes in systolic and diastolic blood pressure from early to mid-gestation

^c^BP changes until mid-gestation	Preeclampsia total			
	n	%	Crude OR (95% CI)^d^	Adjusted^e^ OR (95% CI)
Systolic BP^c^				
Lowered^a^	40,285	3.4	1.0	1.0
Elevated^b^	24,200	3.6	1.1 (1.0–1.1)	1.3 (1.2–1.5)
Diastolic BP^c^				
Lowered^a^	46,473	3.2	1.0	1.0
Elevated^b^	18,009	4.2	1.3 (1.2–1.4)	1.8 (1.6–2.0)

^c^BP: Blood pressure. The changes in systolic and diastolic BP were categorized in the following groups; a) lowered BP, defined as no change or a decrease by 1 mmHg or more, and b) elevated BP, defined as an increase by 1 mmHg or more between early to mid-gestation. ^d^OR (95% CI): Odds ratios with 95% confidence intervals. ^e^Adjusted for mean arterial blood pressure in early gestation, maternal body mass index, smoking, age, recurrent miscarriages before the index pregnancy and country of birth

**Table 3:** Risk of giving birth to small-for-gestational-age (SGA) infants by changes in systolic and diastolic blood pressure from early to mid-gestation

^c^BP changes until mid-gestation	SGA births total			
	n	%	Crude OR (95% CI)^d^	Adjusted^e^ OR (95% CI)
Systolic BP^c^				
Lowered^a^	40,285	3.1	1.0	1.0
Elevated^b^	24,200	2.9	0.9 (0.9–1.0)	1.0 (0.9–1.1)
Diastolic BP^c^				
Lowered^a^	46,473	2.8	1.0	1.0
Elevated^b^	18,009	3.5	1.2 (1.1–1.4)	1.3 (1.2–1.5)

SGA defined as birth weight of more than two standard deviations below the mean weight for gestational age. ^c^*BP* Blood pressure. The changes in systolic and diastolic BP were categorized in the following groups; a) lowered BP, defined as no change or decrease by 1 mmHg or more and b) elevated BP, defined as an increase by 1 mmHg or more between early to mid-gestation. ^d^OR (95% CI): Odds ratios with 95% confidence intervals. ^e^Adjusted for mean arterial blood pressure in early gestation, maternal body mass index, smoking, age, recurrent miscarriages before the index pregnancy and country of birth

For women with lowered diastolic BP from early to mid-gestation, 3.2% were later diagnosed with preeclampsia (Table 2). In women with elevated diastolic BP, the corresponding rate was 4.2%. Compared to women with lowered diastolic BP, women with elevated diastolic BP were at increased risk of preeclampsia, adjusted OR (95% CI); 1.8 [1.6–2.0]. Similarly, the rate of SGA infants was 2.8% in women with lowered diastolic BP and 3.5% in women with elevated diastolic BP (Table 3). Compared to women with lowered diastolic BP, women with elevated diastolic BP were at increased risk of SGA birth, adjusted OR (95% CI); 1.3 [1.2–1.5]. After exclusion of women who developed preeclampsia, the risk increase for SGA in women with elevated diastolic BP remained.

The highest rate of preeclampsia (9.9%) was seen in women with a combination of early-gestation stage 1 hypertension and elevated diastolic BP towards mid-gestation (Table 4). The risk of preeclampsia was three-fold in this group, adjusted OR (95% CI); 3.4 [2.6–4.4], compared to the women with normal early-gestation BP and lowered diastolic BP towards mid-gestation.

**Table 4:** The risk of preeclampsia by stage 1 hypertension in early gestation and changes in systolic and diastolic blood pressure from early to mid-gestation

^c^BP changes until mid-gestation	Preeclampsia total			
	Early gestation blood pressure^d^			
	Normal (< 130/80 mmHg) *n* = 56,491		Stage 1 hypertension^e^ *n* = 7996	
	%	Adjusted OR (95% CI)^f^	%	Adjusted OR (95% CI)^f^
Systolic BP^c^				
Lowered^a^	2.8	1.0	6.5	1.9 (1.7–2.2)
Elevated^b^	3.2	1.1 (1.0–1.2)	9.0	2.8 (2.3–3.4)
Diastolic BP^c^				
Lowered^a^	2.6	1.0	6.6	2.2 (1.9–2.5)
Elevated^b^	3.9	1.4 (1.3–1.6)	9.9	3.4 (2.6–4.4)

^c^*BP* Blood pressure. The changes in systolic and diastolic BP from early to mid-gestation were categorized in the following groups; a) lowered BP, defined as no change or decrease by 1 mmHg or more and b) elevated BP, defined as an increase by 1 mmHg or more between early to mid-gestation. ^d^Blood pressure before 16 weeks. ^e^Stage 1 hypertension: Systolic BP 130–139 mmHg or diastolic BP 80–89 mmHg.^f^Adjusted OR (95% CI): Odds ratios with 95% confidence intervals, adjusted for maternal body mass index, smoking, age, recurrent miscarriages before the index pregnancy and country of birth

Compared with women with lowered diastolic BP from early to mid-gestation, the risks of preterm preeclampsia and preterm SGA increased in women with elevated diastolic BP, with adjusted ORs (95% CI); 2.2 [1.8–2.8] and 2.3 [1.8–3.0], respectively (Table 5).

**Table 5:** The risk of preterm preeclampsia and preterm births of small-for-gestational-age infants (SGA) by changes in diastolic blood pressure from early to mid-gestation

^c^BP changes until mid-gestation	Preterm preeclampsia (< 37 weeks)			
	n	%	Crude OR (95% CI)^d^	Adjusted^e^ OR (95% CI)
Diastolic BP^c^				
Lowered^a^	46,466	0.6	1.0	1.0
Elevated^b^	18,009	0.9	1.6 (1.3–1.9)	2.2 (1.8–2.8)
	Preterm SGA (< 37 weeks)			
	n	%	Crude OR (95% CI)^d^	Adjusted^e^ OR (95% CI)
Diastolic BP^c^				
Lowered^a^	46,410	0.5	1.0	1.0
Elevated^b^	17,980	0.8	1.7 (1.4–2.1)	2.3 (1.8–3.0)

^c^BP: Blood pressure. The changes in systolic and diastolic BP were categorized in the following groups; a) lowered BP, defined as no change or decrease by 1 mmHg or more and b) elevated BP, defined as an increase by 1 mmHg or more between early to mid-gestation. SGA defined as birth weight of more than two standard deviations below the mean weight for gestational age. ^d^OR (95% CI): Odds ratios with 95% confidence intervals. ^e^Adjusted for mean arterial blood pressure in early gestation, maternal body mass index, smoking, age, recurrent miscarriages before the index pregnancy and country of birth

The increase in risk for preterm SGA remained after exclusion of women who developed preeclampsia, adjusted OR (95% CI); 1.8 [1.2–2.6] (data not shown in table).

## Discussion

In this study, we found that elevated diastolic BP from early to mid-gestation was associated with increased risks of preeclampsia and SGA births. The associations seemed stronger for preterm preeclampsia and SGA. This may suggest that elevated BP from early to mid-gestation in contrast to mid-gestation BP drop could be interpreted as a sign of hemodynamic maladaptation to pregnancy in women who later develop placental dysfunction disorders. The results also confirm that a second BP measurement around mid-gestation seems relevant in the antenatal risk assessment [24].

A major strength of the study was the large amount of detailed information enabling us to detect a small effect size and to stratify by early-gestation BP. Being able to define and exclude a risk group of women with suspected vascular disease in early pregnancy [1–3] made it possible to study the effects of BP changes within a cohort of seemingly cardiovascular healthy women. However, the lack of information about family history of preeclampsia and cardiovascular disease may be a limitation in this respect [20]. Information was available on important covariates that may confound the association, such as early pregnancy BMI [19]. Socioeconomic factors may be important confounders for the associations between BP changes during pregnancy and preeclampsia and SGA births [25]. We did not have information on maternal education level or income, but this may have been partly adjusted for by the other socioeconomic-related factors, including country of birth, maternal BMI, smoking, and age at the first pregnancy. The mean gestational age at the mid-gestation BP measurement in this study was 22 gestational weeks, while the nadir of BP is considered to be around 20 weeks [5]. Therefore, we cannot exclude a BP drop before the time-point of measurement in some women categorized with elevated BP from early to mid-gestation that may weaken the associations seen in this study. Further, the register only contains a single BP value for each time-point, and it is therefore unknown to what degree BP was measured three times in a standardized way to obtain an average BP. Although BP measurements are known to be subject to considerable variance, this is mainly expected to negatively impact the power to discriminate between health and disease rather than introduce bias.

In this study, we found increased risks of preeclampsia and SGA in women with elevated diastolic BP from early gestation (mean gestational age 10 weeks) to mid-gestation (mean 22 weeks). A weak association was also found between elevated systolic BP and preeclampsia, but the association was not significant for the outcome of SGA. Macdonald-Wallis et al. found that the risk of preeclampsia increased with increasing diastolic BP from gestational week 18 to 30, while a significant association between a rise in systolic BP and preeclampsia was first seen from week 30 onwards [10]. This study confirms previous findings and suggests that there is an association between rise in BP and risk of preeclampsia at an earlier gestational age than previously observed. In another study by Macdonald-Wallis et al., increases in systolic and diastolic BPs from gestational week 18 to 30 were negatively associated with birth weight and gestational age, and this was also applicable to women who were normotensive during the pregnancy [11]. The approach in our study was different because an association was shown between BP increase and SGA, defined as birth weight for gestational age more than two standard deviations below the population mean. The results confirm an association between diastolic BP changes and birth weight and with preterm SGA infants having very low birth weight.

Interestingly, our results showed that the rates of preeclampsia were almost comparable in women with early-gestation stage 1 hypertension that failed to decrease towards mid-gestation (around 10%) and in the excluded group of women with suspected vascular disease in early gestation (12%). This supports the importance of the early-gestation BP regarding risks of preeclampsia and implies that stage 1 hypertension in early gestation that persists at mid-gestation may be a risk factor for preeclampsia. Future studies are needed to reveal whether women with stage 1 hypertension before mid-gestation in combination with other known risk factors, such as pre-gestational diabetes, may benefit from aspirin [26–28].

The gestational hemodynamic adaptations before mid-gestation include decreased BP [5, 6] and total vascular resistance [7]. These adaptive changes might benefit placental perfusion [8]. The normal BP increase from mid-gestation and onward may reflect an increasing production of vasoconstrictive placental agents, perhaps because of the increased size of the placenta [29] or the increasing cardiovascular demands of pregnancy [30, 31]. Hypoperfusion of the placenta is known to induce production of such vasoconstrictive agents [32, 33], and the production of these agents seems especially high in women developing preeclampsia and intrauterine growth restriction [34]. In this study, we showed an association between elevated BP from early to mid-gestation and preeclampsia and birth of SGA infants. This suggests that the placentas of women experiencing preeclampsia and SGA may already be hypoperfused by mid-gestation because of the successive production of vasoconstrictive agents and increased BP [34]. What causes this placental hypoperfusion is still debated, but a recent hypothesis states that hemodynamic maladaptation to pregnancy may contribute [30]. However, the pathophysiological inference of this study is limited by the lack of pre-gestational hemodynamic measurements and information on adaptive changes before the placenta perfusion is established.

## Conclusion

Our study indicates that the diastolic BP may start to increase before 25 weeks of gestation in women who develop placental dysfunction disorders, especially in women delivering preterm. The results of this study could also imply that both the cardiovascular state prior to pregnancy (reflected by BP in early gestation) and the gestational hemodynamic adaptations during pregnancy (reflected by the change in BP) are related to the pathophysiology of preeclampsia.

## Abbreviations

AaOR: Adjusted odds ratio
BMI: Body mass index
BP: Blood pressure
CI: Confidence interval
ICD-10: International classification of diseases, tenth revision
SGA: Small-for-gestational-age
